# Thermal Performance Optimization of Integrated Microchannel Cooling Plate for IGBT Power Module

**DOI:** 10.3390/mi14081498

**Published:** 2023-07-26

**Authors:** Hanyang Xu, Jiabo Huang, Wenchao Tian, Zhao Li

**Affiliations:** School of Electro-Mechanical Engineering, Xidian University, Xi’an 710071, China; 20041211862@stu.xidian.edu.cn (J.H.); wctian@xidian.edu.cn (W.T.); zhaoli960628@163.com (Z.L.)

**Keywords:** IGBT power module, microchannel, cooling plate, micropump

## Abstract

In high-integration electronic components, the insulated-gate bipolar transistor (IGBT) power module has a high working temperature, which requires reasonable thermal analysis and a cooling process to improve the reliability of the IGBT module. This paper presents an investigation into the heat dissipation of the integrated microchannel cooling plate in the silicon carbide IGBT power module and reports the impact of the BL series micropump on the efficiency of the cooling plate. The IGBT power module was first simplified as an equivalent-mass block with a mass of 62.64 g, a volume of 15.27 cm^3^, a density of 4.10 g/cm^3^, and a specific heat capacity of 512.53 J/(kg·K), through an equivalent method. Then, the thermal performance of the microchannel cooling plate with a main channel and a secondary channel was analyzed and the design of experiment (DOE) method was used to provide three factors and three levels of orthogonal simulation experiments. The three factors included microchannel width, number of secondary inlets, and inlet diameter. The results show that the microchannel cooling plate significantly reduces the temperature of IGBT chips and, as the microchannel width, number of secondary inlets, and inlet diameter increase, the junction temperature of chips gradually decreases. The optimal structure of the cooling plate is a microchannel width of 0.58 mm, 13 secondary inlets, and an inlet diameter of 3.8 mm, and the chip-junction temperature of this structure is decreased from 677 °C to 77.7 °C. In addition, the BL series micropump was connected to the inlet of the cooling plate and the thermal performance of the microchannel cooling plate with a micropump was analyzed. The micropump increases the frictional resistance of fluid flow, resulting in an increase in chip-junction temperature to 110 °C. This work demonstrates the impact of micropumps on the heat dissipation of cooling plates and provides a foundation for the design of cooling plates for IGBT power modules.

## 1. Introduction

Insulated-gate bipolar transistor (IGBT) power modules are widely used in high-power-converter applications [1,2]. As the integration of IGBT modules gradually increases, the voltage and power level also increase, leading to an increase in the heating of electronic components [3]. Normally, the maximum operating junction temperature of IGBT power modules is 125 °C and the operating junction temperature of silicon carbide (SiC) power modules is higher, reaching to over 200 °C [4,5]. However, excessive junction temperatures or severe temperature fluctuations in IGBT power modules can lead to problems such as boundary warping [6], solder delamination [7], gate oxide layer damage [8], and gate spring pin fatigue failure [9] in electronic components. Hence, it is necessary to use effective heat dissipation methods to reduce the device temperature so that the chip temperature is stable and lower than the maximum allowable junction temperature.

The heat transfer process of the IGBT module includes the power loss of the IGBT chip, the temperature conduction of the chip to the IGBT module shell, the heat conduction to the radiator of the IGBT module, and the thermal conduction from the radiator to the air [10]. Therefore, the operating temperature of IGBT power modules can be reduced by reducing the power loss of IGBT chips and the thermal resistance of radiators [11,12]. However, due to the limitations of output power and the actual operating conditions of IGBT modules, the total loss of IGBT cannot be changed and radiators are the main trending method to reduce the operating temperature of the IGBT power module [13]. The heat dissipation methods of IGBT devices include wind and water cooling [14]. Compared with wind-cooling methods, the microchannel liquid-cooling method has the advantages of a compact structure, low noise, and high heat efficiency [15,16]. In addition, microchannel structures can be designed for different heat sources and the chip substrate temperature is lower and the temperature distribution is more uniform [17]. In 2020, Mohanad studied the heat transfer characteristics of a microchannel cooling plate and explored the influence of inclined grooves with different heights and angles on cooling efficiency [18]. The results show that the heat dissipation capacity of the cooling plate increases with the height and inclination angle of the inclined grooves. Regarding the heat dissipation capacity of microchannels with different structures, Liming Yin designed a microchannel radiator with a T-shaped coolant distributor [19]. The results show that the chip temperature was reduced by 7.4 °C compared to conventional microchannel radiators. The effects of microchannel materials [20], cooling liquid [21], and the geometric parameters of the microchannel [22] on temperature distribution, pressure drop, and thermal resistance of microchannel cooling plates have been studied in previous research. However, reporting on the impact of micropumps on the heat dissipation efficiency of microchannel cooling plates is rare. Further research is needed to determine the thermal performance of the liquid-cooling method, especially for IGBT power modules with higher working temperatures.

This paper presents a numerical study of the microchannel heat dissipation cooling plate for an IGBT power module. The IGBT power module was made up of 16 SiC power chips and was simplified by equivalent thermal resistance conditions. The orthogonal simulation experimental method was used to analyze the effects of microchannel width, number of secondary inlets, and inlet diameter on the heat dissipation efficiency of microchannel cooling plates, and the optimal structure of microchannel cooling plates was obtained. In addition, considering the frictional resistance of the micropump and the connecting pipe, the micropump installation scheme was designed and the model with a micropump installed was simulated to obtain the chip-junction temperature. This research provides a foundation for the integrated design of microchannel cooling plates for IGBT power modules.

## 2. Computational Models and Methods

### 2.1. Geometric Modeling of IGBT Power Module

**Figure 1:**
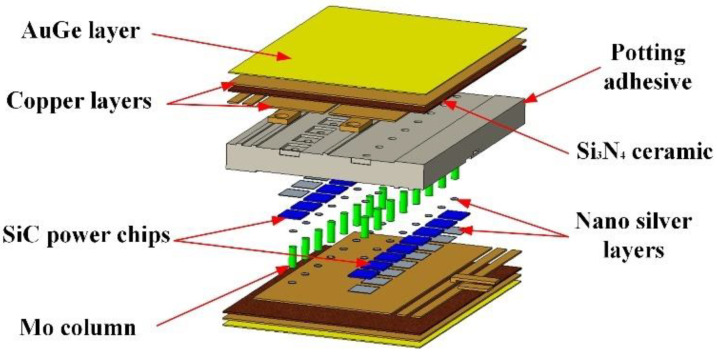
Schematic diagram of IGBT power module.

Figure 1 shows the IGBT power module in this work. The IGBT power module on the high-temperature cofired ceramic (HTCC) substrate consists of 16 chips. (The internal resistance of the chip is 40 mΩ, the conduction current is 40 A, and the duty cycle is 50%).

The IGBT power module is divided into two parts: the upper and lower bridges. Each bridge includes eight SiC power chips, nanosilver layers, direct-bond copper (DBC) plate, a gold–germanium (AuGe) layer, molybdenum columns (Mo columns), and potting adhesive. The DBC plate is composed of two layers of copper and silicon nitride ceramic (Si_3_N_4_ ceramic). The bottom of the SiC power chip is connected to the copper layer of the DBC plate through a nanosilver layer, and the top is connected to the Mo column through a nanosilver layer. The Mo column connects the SiC power chip with the DBC plate of the other half bridge. The AuGe layer is mainly used for connecting DBC plates and cooling plates in the IGBT power module.

The effort of modeling and simulation calculation is substantial due to the complexity of the IGBT power module. Hence, the equivalent method was used in this research to simplify IGBT devices and the temperature of SiC chips is the major emphasis [23]. The equivalent method needs to meet the following conditions:

(1) The size and quality are the same before and after equivalency;
(2) The effect of the model before and after equivalency on-chip heat dissipation is the same. (This signifies that the thermal conductivity of the equivalent block is the same as the thermal conductivity of the actual model).

First, simplify the geometric dimensions of the IGBT power module. The influence of density and specific heat capacity on heat is linear. Hence, the mass of the equivalent-mass block can be determined by the sum of the masses of each part and the density of the equivalent-mass block can be determined by dividing the mass of the equivalent-mass block by the total volume of the equivalent-mass block. The geometric size parameters of different structures are shown in Table 1. According to the measurement of the different materials’ volumes using 3D-modeling software, the total volume of equivalent-mass blocks is obtained by adding the volumes of the different materials. The calculation results are shown in Table 2. (Some material parameters are obtained through experimental testing, such as the density and specific heat capacity of the SiC chips.) The equivalent total mass is 62.64 g, the equivalent total volume is 15.27 cm^3^, and the equivalent-mass block density is 4.10 g/cm^3^. The calculation method for the specific heat capacity of the equivalent-mass block is to multiply the volume of each structure by its respective specific heat capacity and divide it by the total volume after accumulation. The specific heat capacity of the equivalent mass is 512.53 J/(kg·K).

Secondly, determine the equivalent thermal conductivity of the simplified IGBT power module. The equivalent thermal conductivity *K* can be calculated through equivalent thermal resistance.

(1) K=h/(R⋅A)

where *h* is the height of the heat transfer direction, *A* is the cross-sectional area of the heat transfer direction, and *R* is the thermal resistance of the heat transfer direction.

IGBT power module heat transfer modes include vertical and horizontal heat transmission. Therefore, the equivalent thermal conductivity in the vertical direction and the equivalent thermal conductivity in the horizontal direction must be calculated independently. Table 3 shows the height in the vertical direction, the cross-sectional area in the vertical direction, and the thermal conductivity of various structures.

Thermal resistance series or parallel connection works on the same principle as resistance series or parallel connection. In the vertical direction, the Mo column is connected in series with the nanosilver layer for thermal conductivity. After being connected in series with the nanosilver layer, the Mo column is connected in parallel with the high thermal conductivity potting adhesive and then connected in series with copper, Si_3_N_4_, and AuGe. The thermal conductivity of the equivalent-mass block in the vertical direction is 8.33 W/(m·K).

In the horizontal direction, the Mo column and nanosilver layer are connected in parallel for thermal conductivity. The Mo column and nanosilver layer are connected in series with the potting adhesive and then connected in parallel with copper, Si_3_N_4_, and AuGe. In addition, the heat transfer height *h* in the horizontal direction of these structures is the same and the heat transfer cross-sectional area is directly proportional to the heat transfer height *h* in the vertical direction. The thermal conductivity of the equivalent-mass block in the horizontal direction is 105.47 W/(m·K).

Finally, the equivalent model was coded in COMSOL software and the initial conditions, boundary conditions, and material properties of the equivalent model were the same as the original device model. Convective heat transfer was set on the outer surface of the device (the convective heat transfer coefficient is 10 W/(m^2^·°C)). In addition, the chips were set to work alternately on the upper and lower bridges, each working for 300 s, with a cycle of 600 s, and the volume heat being 3.87 × 10^9^ W/m^3^. The temperature fields of the original IGBT device and the equivalent device were simulated separately to obtain the highest temperature of the chips.

**Figure 2:**
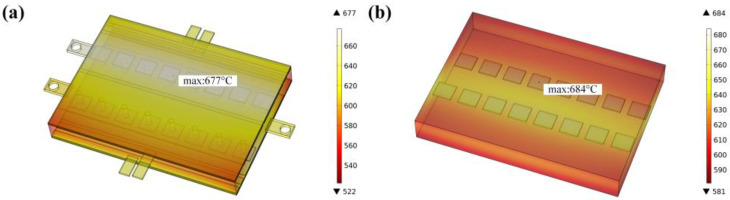
Schematic diagram of temperature-field simulation results for IGBT power module; (**a**) is the temperature distribution results of the original module and (**b**) is the temperature distribution results of the equivalent module.

The temperature-field simulation results are shown in Figure 2. Figure 2a shows the temperature field of the original device chip and the maximum temperature is 677 °C. Figure 2b shows that the maximum temperature of the equivalent device chip is 684 °C. The difference between the highest temperature of the equivalent device chip and the highest temperature of the original device chip is 1.0%. Therefore, this equivalent method can be adopted.

### 2.2. Geometric Modeling of Cooling Plate

The internal flow channels of the cooling plate can be divided into series flow channels and parallel flow channels based on their distribution [26]. As there is only one channel in the series flow channel, the flow velocity inside the channel is higher while the inlet flow rate is constant, increasing the convective heat transfer coefficient. However, the length of the series flow channel is much longer than the length of the parallel flow channel and the thickness of the boundary layer increases with the increase of the channel length, affecting the effectiveness of convective heat transfer [27]. Moreover, the frictional resistance of the series flow channel is relatively high, the heat transfer efficiency in the latter half of the channel is less, and the requirements for the system pump power are higher. The parallel flow channel is composed of multiple parallel flow channels. Compared to the series flow channel, the parallel flow channel has lower frictional resistance, which helps to increase heat transfer efficiency [28,29].

**Figure 3:**
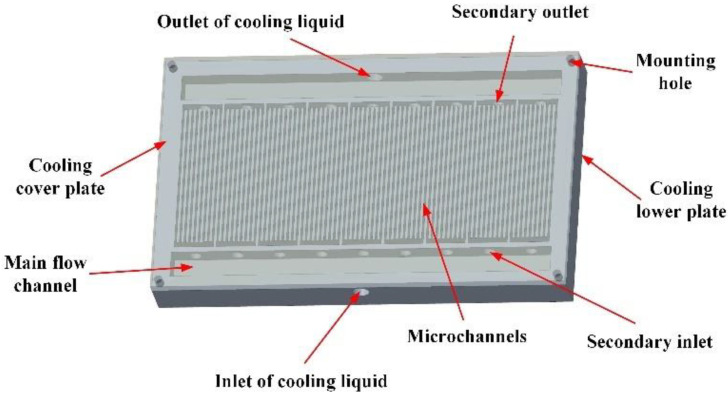
Schematic diagram of the three-dimensional structure of the cooling plate.

In this work, the IGBT power module is small in geometry and a smaller micropump drive is required for easy installation and normal operation of the device. Therefore, the cooling plate was designed as a parallel flow channel with low pump-power requirements. The cooling plate consists of a cooling lower plate and a cooling cover plate, where the size of the cooling lower plate is 80 mm × 47.6 mm × 8 mm, and the microchannel depth is 4 mm. The side of the cooling lower plate is equipped with an inlet and outlet for cooling liquid. In addition, the cooling lower plate contains two main flow channels which are connected to the inlet and outlet, respectively. The main flow channels can be used for the secondary distribution of fluids and to improve the defect of uneven flow velocity distribution in parallel flow channels [28]. The microchannel is separated into independent parts by fins, with each fin having a secondary inlet and outlet connected to the main channel. The thickness of the microchannel fins is 0.5 mm, the height is 4 mm, and the distance between the two fins is 0.4 mm. The size of the cooling cover plate is 80 mm × 47.6 mm and the thickness is 2 mm. Figure 3 shows the three-dimensional model of the cooling plate.

Two cooling plates were installed on the upper and lower surfaces of the IGBT power module, respectively, in order to improve the heat dissipation effect of the cooling plate. The cooling plate was connected to the power module using an AuGe layer. In addition, the overall structure was installed using an aluminum shell.

### 2.3. Governing Equations

During the heat transfer process, the Reynolds number does not reach the critical value of turbulence (less than 150) Therefore, the Navier–Stokes equation for incompressible laminar flow was used to solve the flow velocity and pressure change in the fluid flow.

(2) $$\rho \left(\frac{\partial u}{\partial \tau }+u\frac{\partial u}{\partial x}+v\frac{\partial u}{\partial y}\right)=Fx-\frac{\partial p}{\partial x}+\eta \left(\frac{\partial^{2}u}{\partial x^{2}}+\frac{\partial^{2}u}{\partial y^{2}}\right)$$

(3) $$\rho \left(\frac{\partial v}{\partial \tau }+u\frac{\partial v}{\partial x}+v\frac{\partial v}{\partial y}\right)=Fy-\frac{\partial p}{\partial y}+\eta \left(\frac{\partial^{2}v}{\partial x^{2}}+\frac{\partial^{2}v}{\partial y^{2}}\right)$$

where, *ρ* is the density, *u* and *v* are the velocity components in the X and Y directions, respectively, *P* is the pressure, *Fx* and *Fy* are the external force components in the X and Y directions, respectively, and *η* is the viscosity.

The mass diffusion in the flow channel can be described by the continuity equation:

(4) $$\frac{\partial u}{\partial x}+\frac{\partial v}{\partial y}=0$$

The heat transfer process of the IGBT power module includes internal heat conduction, forced convection heat transfer, and fluid–solid coupling heat transfer. The energy conservation equation for the solid phase is shown below:

(5) $$\nabla \cdot \left(-\lambda_{s}^{}\nabla T\right)=Q_{heat}$$

where, *λ_s_* is the thermal conductivity of solids and *Q_heat_* is the chip heat source.

The energy conservation equation for the liquid phase is shown below:

(6) $$\rho C_{p}u\cdot \nabla T+\nabla \cdot \left(-\lambda_{l}^{}\nabla T\right)=0$$

where, *C_p_* is the specific heat capacity of fluids, *λ_l_* is the thermal conductivity of liquids.

### 2.4. Computational Models and Boundary Conditions

**Figure 4:**
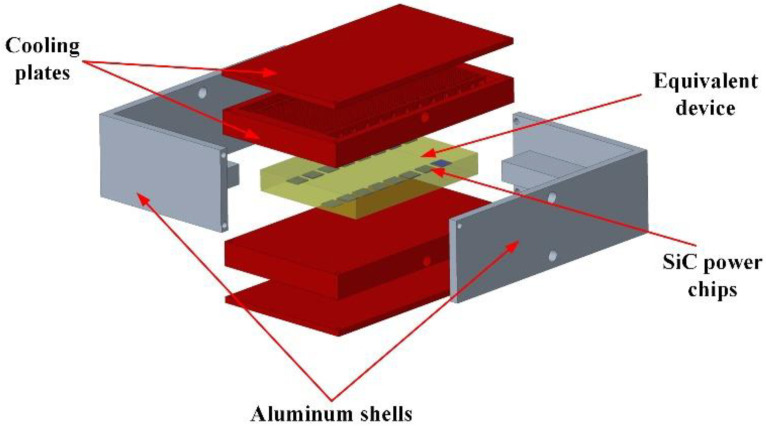
Schematic diagram of IGBT power module and cooling plate structure.

The numerical model was coded in COMSOL software. The overall installation model is shown in Figure 4. The model consisted of aluminum shells, the equivalent devices, 16 SiC power chips, and two cooling plates.

Table 4 shows the properties of materials. The materials of the cooling lower plate and cooling cover plate are copper, the material of the SiC power chips is silicon carbide, and the material of the cooling liquid is water.

The boundary conditions of the model are shown below:

(1) For the module of incompressible laminar flow, the pressure at the inlet of the fluid is 200 Pa, the pressure at the outlet is 0 Pa, and the initial temperature of the cooling liquid is 20 °C;
(2) For the module of heat transfer, the heat sources of 16 SiC chips are 3.87 × 10^9^ W/m^3^;
(3) The condition of the walls in incompressible laminar flow module is no slipping wall.

Heat transfer mainly occurs inside the microchannel and at the contact surfaces between the chips and the cooling plate. Therefore, special mesh refinement processing was required for these regions. A mesh consisting of 8,731,096 elements was generated using the free tetrahedral meshing method, and the average cell quality of the mesh reached 0.6.

## 3. Result and Discussion

### 3.1. Thermal Performance of Microchannel Cooling Plate

**Figure 5:**
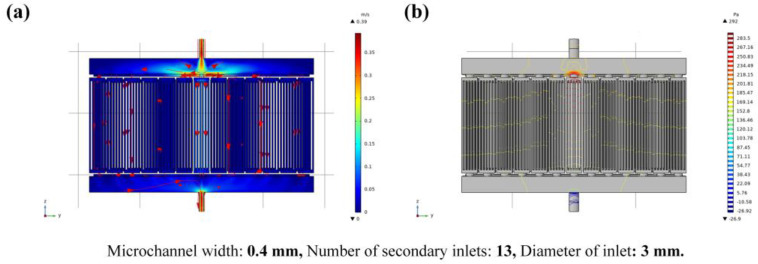
Fluid schematic diagram of the cooling plate; (**a**) is the cloud chart of fluid velocity distribution and (**b**) is the cloud chart of fluid pressure distribution.

Figure 5 shows the fluid schematic diagram of the cooling plate and the flow velocity distribution cloud diagram is shown in Figure 5a. Due to the influence of flow friction resistance, the velocity of the fluid will gradually decrease with the flow of the fluid and the velocity of the fluid away from the inlet is smaller than that of the fluid at the inlet. Here, the velocity at the inlet of the fluid is the highest (0.39 m/s). In addition, as shown in Figure 5b, the pressure of the fluid in the middle channel and near the inlet is relatively high, while the pressure in the edge channels and near the outlet is relatively low. The maximum pressure in the model is 292 Pa.

**Figure 6:**
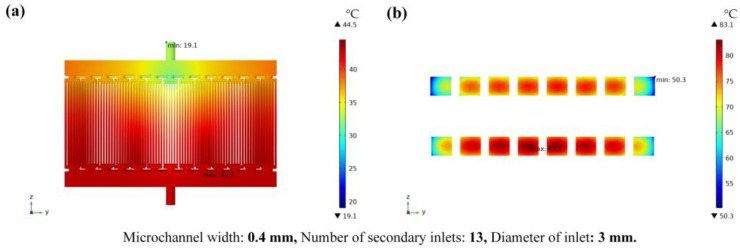
Schematic diagram of the temperature results; (**a**) is the cloud chart of fluid temperature distribution and (**b**) is the cloud chart of chips temperature distribution.

Figure 6 shows the temperature results. The temperature distribution cloud diagram of the fluid part is shown in Figure 6a; the maximum temperature of the fluid part is 44.5 °C. The temperature distribution result of the chips is shown in Figure 6b. Here, the temperature of the chip near the lower middle position is highest at 83.1 °C. In addition, the temperature of the middle chip is higher than that of the chips on both sides. The chip located in the middle and near the outlet of the heat dissipation cooling plate emits less heat due to its shorter heat dissipation path, resulting in the highest temperature.

The number of grading inputs, the size of the input, and the width of the microchannel will cause the velocity of the cooling liquid away from the inlet to be lower than the velocity of the inlet point. Meanwhile, the temperature of the chips shows a lower temperature on both sides and a higher temperature in the middle as the fluid velocity changes. Therefore, the structure of the microchannel and the structure of the inlet have a significant impact on the heat transfer performance of the microchannel and a reasonable geometric design of the microchannel is the key to heat transfer enhancement.

In order to find the optimal microchannel structure for heat dissipation, the cooling plate structure was optimized. The maximum junction temperature of chips was used as an indicator and the width of the microchannel, number of secondary inlets, and diameter of the inlet were used as design variables to determine the range of various parameters. Design of experiment (DOE) was used in this work to provide orthogonal simulation experiments with three factors and three levels. For the convenience of calculation, the three factors were represented by A, B, and C, and the three levels of each factor were represented by one, two, and three, respectively. For example, A_1_ represents a microchannel width of 0.4 mm. The results of the orthogonal simulation experiment are shown in Table 5:

It can be seen from Table 5 that the lowest junction temperature of chips was number 7 in the nine orthogonal simulation experiments, and the maximum junction temperature of chips was 77.7 °C. However, the orthogonal simulation experiments were only 1/64 of all combinations (the number of all combinations is 4^5^ = 1024), and the optimal combination obtained through orthogonal simulation experiments may not necessarily be the best of all combinations. Therefore, it is necessary to analyze the simulation results and find the theoretically optimal combination.

The range method was used to analyze the orthogonal simulation results. Firstly, the maximum junction temperature of chips for several repeated experiments at each level is summed and represented as *K_i_* (*i* represents the different levels of each factor). In addition, the average value of the maximum junction temperature of chips is called the average value of the indicator and is represented by $\bar{K_{i}}$. The optimal level of factors can be obtained through *K_i_* and $\bar{K_{i}}$.

Secondly, to compare the experimental indicators of different factors at different levels, the range *R* was defined to describe the significance of each factor in the orthogonal simulation experiment. The range *R* is the difference between the maximum and minimum $\bar{K_{i}}$ values of each factor at each level, as shown in Equation (7).

(7) $$R={\bar{K_{i}}}_{\max}-{\bar{K_{i}}}_{\min}$$

Finally, based on the simulation results of the maximum junction temperature of chips in nine devices, the sum, the average, and the range of indicators were obtained as shown in Table 6.

**Figure 7:**
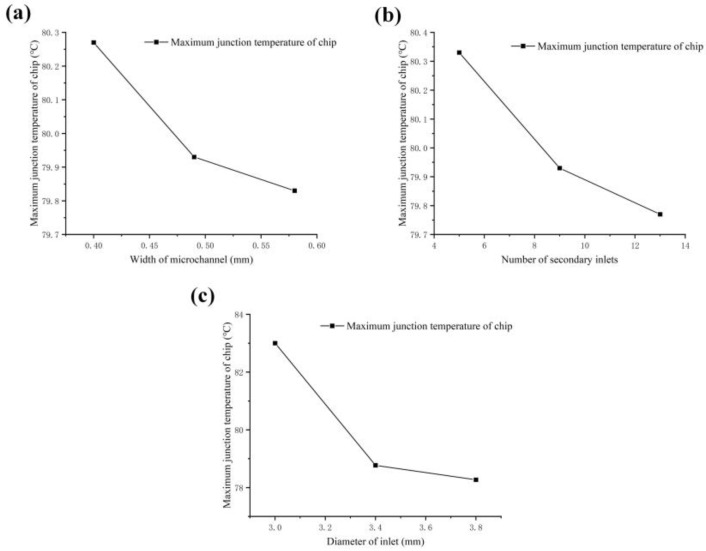
Schematic diagram of the relationship between three factors and chip-junction temperature; (**a**) is the width of the microchannel, (**b**) is the number of secondary inlets, and (**c**) is the diameter of the inlet.

The amount of the range reflects the degree to which factors affect the experimental indicators. A large range value indicates that the factor has a significant impact on the indicator and is called the main factor. A small range value indicates a small impact on the indicator and is referred to as a secondary factor. Figure 7 shows the influence of the average values of different factors on the maximum junction temperature of the chips.

Figure 7a shows the effect of microchannel width on the maximum junction temperature of the chips. Within the range of 0.40~0.60 mm, as the width of the microchannel increases, the maximum junction temperature of the power chip gradually decreases, with the maximum temperature of the chip decreasing from 80.27 °C to 79.83 °C. The main reason is that as the width of the microchannel increases, the fluid flow rate increases, the convective heat transfer efficiency increases, and the maximum junction temperature of the chips decreases.

Figure 7b shows the effect of the number of secondary inlets on the maximum junction temperature of the chips. As the number of secondary inlets increases, the highest junction temperature of the chip gradually decreases and the junction temperature decreases from 80.33 °C to 79.77 °C. This is due to the greater the number of secondary inputs, the more uniform the flow rate of the fluid, and the higher the efficiency of heat exchange.

Figure 7c shows the effect of the diameter of the inlet on the maximum junction temperature of the chips. As the diameter of the water inlet decreases, the highest temperature of the chip decreases, and the temperature of the chip decreases from 83 °C to 78.27 °C. This is because as the diameter of the inlet increases, the flow rate of the fluid increases, and the fluid carries more heat away, resulting in higher heat transfer efficiency.

It can be seen from Figure 7 that A_3_ has the lowest temperature among the three levels of factor A (width of the microchannel), B_1_ has the lowest temperature among the three levels of factor B (number of the secondary inlets), and C_3_ has the lowest temperature among the three levels of factor C (diameter of the inlet). Therefore, the theoretical optimal level combination is A_3_B_1_C_3_, which is the cooling plate with a microchannel width of 0.58 mm, 13 secondary channels, and an inlet diameter of 3.8 mm. In addition, the order of influence of the three factors is the diameter of the inlet > number of secondary inlets > width of the microchannel.

**Figure 8:**
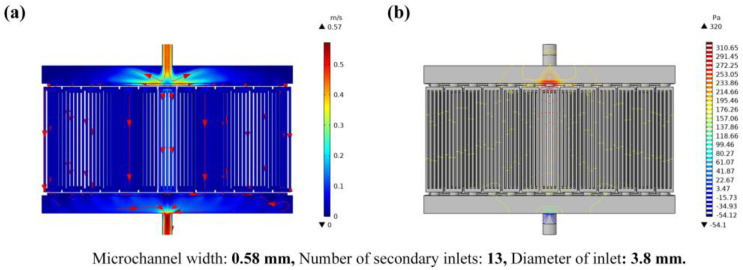
Fluid schematic diagram of the theoretical optimum model; (**a**) is the cloud chart of fluid velocity distribution and (**b**) is the cloud chart of fluid pressure distribution.

**Figure 9:**
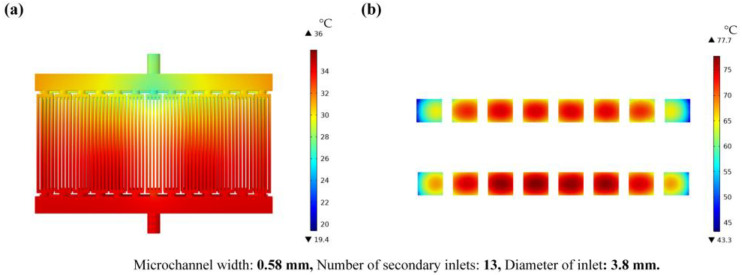
Temperature schematic diagram of the theoretical optimum model; (**a**) is the cloud chart of fluid temperature distribution, (**b**) is the cloud chart of chip temperature distribution.

Figure 8 and Figure 9 show the fluid flow velocity, pressure, fluid temperature, and chip temperature results of the theoretical optimum model (A_3_B_1_C_3_). The flow-velocity distribution diagram of the fluid and the fluid-pressure distribution diagram are shown in Figure 8a,b. The maximum flow velocity and the maximum pressure are 0.57 m/s and 320 Pa, respectively. Figure 9a,b show the temperature distribution of fluid and chips. The maximum temperature of the fluid is 36 °C and the maximum temperature of the chip is 77.7 °C.

### 3.2. Effect of Micropumps on the Thermal Performance of Microchannel Cooling Plates

**Figure 10:**
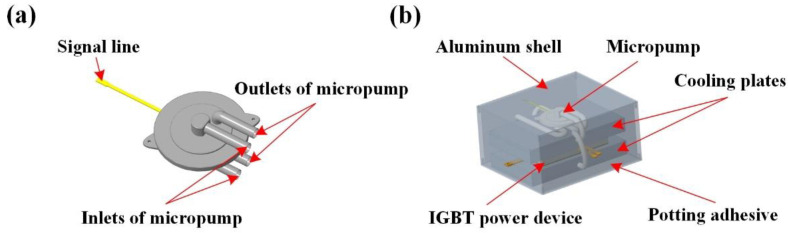
The IGBT power module cooling plate with micropumps; (**a**) is the schematic diagram of the BL series micropump and (**b**) is the installation diagram of cooling plates and micropump.

The experiment for the effect of the micropump (BL series piezoelectric micropump) on the heat dissipation efficiency of cooling plates was designed, as shown in Figure 10. The micropump has two inlet and two outlet ports, which can meet the requirements of the two cooling plates in this work. The diameter of the micropump is 29 mm and the length of the micropump is 11 mm, as shown in Figure 10a. The peak flow rate can be greater than 1.65 L/min and the peak pressure can be greater than 300 mbar.

Based on the dimensional parameters and schematic diagram provided by the micropump manufacturer, a three-dimensional model of the micropump was established. The micropump outlet is connected to the cooling plate inlet, and the cooling plate outlet is connected to the micropump inlet, forming a flow cycle of cooling liquid. In addition, the aluminum shell was installed on the outside of the device, and the device was filled with insulation potting adhesive to protect and secure the device. The installation scheme of the micropump, cooling plates, and device is shown in Figure 10b.

**Figure 11:**
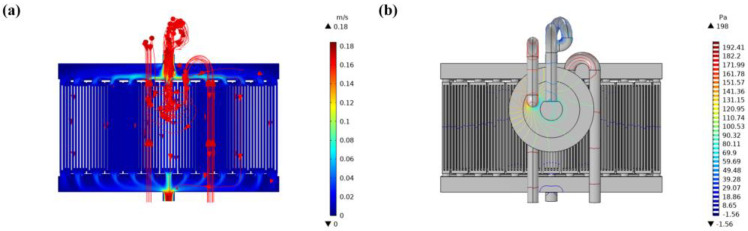
Fluid schematic diagram of the cooling plate model with a micropump; (**a**) is the cloud chart of velocity distribution and (**b**) is the cloud chart of fluid pressure distribution.

In order to analyze the effect of micropumps on the thermal performance of microchannel cooling plates, numerical simulations were conducted using the same cooling plate structure and boundary condition parameters to calculate the heat dissipation effect of the cooling plates with micropumps installed. The structure of the cooling plate was A_3_B_1_C_3_, and the initial inlet pressure was 200 Pa. The velocity and pressure distribution of the cooling liquid in the cooling plate after installing a micropump are shown in Figure 11a,b, respectively. It can be seen from Figure 11 that, due to the installation of the micropump, the fluid resistance along the way increases and the inlet flow rate of the cooling plate fluid decreases. The maximum velocity decreases from 0.57 m/s to 0.18 m/s and the maximum pressure drop changes from 320 Pa to 198 Pa. Besides, the micropump did not change the distribution trend of cooling velocity and pressure field.

**Figure 12:**
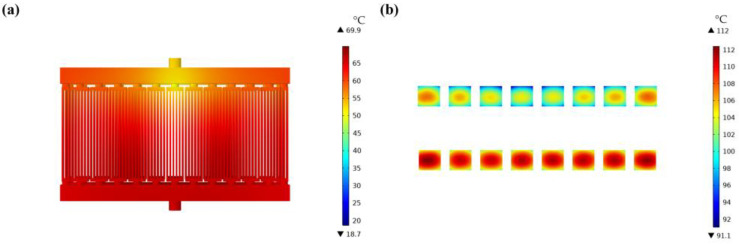
Temperature schematic diagram of the cooling plate model with micropump; (**a**) is the cloud chart of fluid temperature distribution and (**b**) is the cloud chart of chip temperature distribution.

Figure 12a,b show the fluid temperature distribution in the cooling plate after installing the micropump and the schematic diagram of the maximum temperature of the chip, respectively. The micropump causes a decrease in the velocity of the cooling liquid inside the cooling plate, resulting in a decrease in the heat transfer efficiency of the cooling plate. The maximum temperature of the fluid inside the cooling plate has increased from 36 °C (no micropump installation) to 69.9 °C, as shown in Figure 12a, and the maximum temperature of the chip has increased from 77.7 °C (no micropump installation) to 110 °C, as shown in Figure 12b. Table 7 shows the maximum junction temperatures of the original chips, the chips with optimized cooling plates, and the chips with micropump.

In addition, the temperature of the cooling liquid near the inlet of the cooling plate is lower, while the temperature of the cooling liquid near the outlet of the cooling plate is higher, which is the same trend as the fluid temperature without a micropump installed. However, as the micropump increases the distance of fluid flow, the resistance of the fluid along the way increases and the velocity of the cooling plate fluid input decreases. The maximum temperature of the chip increases compared to the maximum temperature without a micropump installed. Hence, it is necessary to appropriately reduce the distance between the micropump and the cooling plate or increase the pressure at the inlet of the cooling plate to meet the heat dissipation temperature requirements of the chips.

## 4. Conclusions

This paper presents a numerical study of a microchannel heat dissipation cooling plate for an IGBT power module and analyzed the thermal performance of a microchannel cooling plate with a micropump. The following conclusions are drawn:

(1) The microchannel cooling plate can reduce the operating temperature of IGBT power modules. In addition, as the width of the microchannel, the number of secondary inlets, and the diameter of the inlet increase, the chip-junction temperature gradually decreases. The order of influence of the three factors is the diameter of the inlet > number of secondary inlets > width of the microchannel;
(2) The optimal structure of the cooling plate theory obtained by orthogonal analysis is that the width of the microchannel is 0.58 mm, the number of secondary inlets is 13, the diameter of inlets is 3.8 mm, and the maximum junction temperature of chips decreased from 677 °C to 77.7 °C. Therefore, the temperature of the chip can be significantly reduced by optimizing the cooling plate structure reasonably;
(3) The micropump increases the distance of fluid flow, the resistance of the fluid along the way increases, and the velocity of the cooling plate fluid input decreases. The maximum chip-junction temperature of the microchannel cooling plate after installing the micropump increased from 77.7 °C to 110 °C. Hence, in order to reduce the impact of micropumps on the cooling efficiency of the cooling plate, the length of the connecting pipe between the micropump and the cooling plate should be reduced.

## Figures and Tables

**Table 1:** Dimensional parameters of different structures.

Structure	Material	Length	Wide	Height	Diameter
SiC chips	SiC	5 (mm)	4.6 (mm)	0.36 (mm)	/
Chip solder layer	Nanosilver	5 (mm)	4.6 (mm)	0.10 (mm)	/
Mo column solder layer	Nanosilver	/	/	0.10 (mm)	1.5 (mm)
Mo column	Mo	/	/	4 (mm)	1.5 (mm)
DBC adhesive layer	AuGe	55 (mm)	40 (mm)	0.20 (mm)	/
Upper layer of DBC	Copper	55 (mm)	40 (mm)	0.3 (mm)	/
DBC ceramic	Si_3_N_4_	55 (mm)	40 (mm)	0.32 (mm)	/
Bottom layer of DBC	Copper	55 (mm)	34 (mm)	0.3 (mm)	/

**Table 2:** Equivalent parameters of geometric dimensions in 20 °C [24,25].

	Density (g/cm^3^)	Specific Heat Capacity (J/(kg·K))	Volume (cm^3^)	Mass (g)
Si_3_N_4_	3.2	710	1.408	4.5056
Copper	8.9	385	2.47303	20.773452
Mo column	10.2	250	0.113097	1.1535894
Nanosilver layer	10.49	232	0.042455	0.44535295
AuGe	17.5	130	0.88	15.4
Potting adhesive	1.95	550	10.2241	19.936995
SiC chip	3.2	670	0.13248	0.423936
**Equivalent device**	**4.10**	**512.53**	**15.27**	**62.64**

**Table 3:** Equivalent table of thermal conductivity coefficient.

Material	Thermal Conductivity (W/(m·K))	*h_vertical_* (mm)	*A_vertical_* (mm^2^)
Si_3_N_4_	80	0.64	2200
Copper	400	1.2	2200
Mo	137	4	28.27
Nanosilver	418	0.1	28.27
AuGe	44.4	0.4	2200
Potting adhesive	4	4.66	2171.73

**Table 4:** The properties of materials.

Materials	Density (kg/m^3^)	Thermal Conductivity (W/(m·K))	Specific Heat Capacity (J/(kg·K))	Dynamic Viscosity (Pa·s)
Copper	8900	400	390	/
SiC	3200	400	670	/
Aluminum	2700	210	880	/
Equivalent devices	4100	8.33 (XY direction) 105.47 (Z direction)	512.53	/
Water	1000	0.609 (25 °C) 0.648 (50) 0.671 (75 °C) 0.683 (100 °C)	4200	9.028 × 10^−4^ (25 °C) 5.494 × 10^−4^ (50 °C) 3.806 × 10^−4^ (75 °C) 2.825 × 10^−4^ (100 °C)

**Table 5:** Results of orthogonal simulation experiments.

Number	Microchannel Width (mm)	Number of Secondary Inlets	Diameter of Inlet (mm)	Maximum Junction Temperature of Chips (°C)
1	A_1_ 0.40	B_1_ 13	C_1_ 3.0	83.1 °C
2	A_1_ 0.40	B_2_ 9	C_2_ 3.4	78.8 °C
3	A_1_ 0.40	B_3_ 5	C_3_ 3.8	78.9 °C
4	A_2_ 0.49	B_1_ 13	C_2_ 3.4	78.5 °C
5	A_2_ 0.49	B_2_ 9	C_3_ 3.8	78.2 °C
6	A_2_ 0.49	B_3_ 5	C_1_ 3.0	83.1 °C
7	A_3_ 0.58	B_1_ 13	C_3_ 3.8	77.7 °C
8	A_3_ 0.58	B_2_ 9	C_1_ 3.0	82.8 °C
9	A_3_ 0.58	B_3_ 5	C_2_ 3.4	79.0 °C

**Table 6:** The sum, average, and range of factors.

	Factor A	Factor B	Factor C
*K*_1_	240.8	239.3	249
*K*_2_	239.8	239.8	236.3
*K*_3_	239.5	241	234.8
$\bar{K_{1}}$	80.27	79.77	83
$\bar{K_{2}}$	79.93	79.93	78.77
$\bar{K_{3}}$	79.83	80.33	78.27
Range	0.44	0.56	4.73
The order of influence of factors	C > B > A		

**Table 7:** Maximum junction temperature of chips with different results.

	Maximum Junction Temperature of Chips
Original chips	677 °C
Chips with optimized cooling plate	77.7 °C
Chips with micropump	110 °C

## Data Availability

Not applicable.
